# The adoption, efficacy, and limitations of large language models in urological education: A cross-sectional survey

**DOI:** 10.1097/MD.0000000000051124

**Published:** 2026-10-16

**Authors:** Wesam Khan

**Affiliations:** a Department of Urology, University of Tabuk, Tabuk, Saudi Arabia.

**Keywords:** artificial intelligence, ChatGPT, clinical reasoning, large language models, medical education, medical training, urology

## Abstract

The integration of large language models into medical education is rapidly transforming pedagogical approaches, particularly in specialized fields like urology. This study aims to evaluate the adoption patterns, perceived efficacy, and significant limitations of these artificial intelligence (AI) tools among urology trainees. This research employed a descriptive, cross-sectional study design through an online survey disseminated to urology trainees at a single institution. A total of 194 complete responses were collected from medical students and interns. The survey assessed adoption patterns, perceived efficacy on a 7-point Likert-scale, and limitations. The analysis revealed near-universal adoption (98.4%), with 65% of participants using AI tools daily, predominantly ChatGPT. Key applications included generating exam-style questions and summarizing complex guidelines. Participants reported significant perceived improvements in core knowledge areas, with the highest gains in disease knowledge and diagnostics (Median = 7, interquartile range: 6–7), which were strongly correlated with increased clinical confidence. Multivariate regression identified usage frequency and comfort with technology as significant predictors of knowledge improvement (β = 0.29, *P* = .001) and confidence gains (β = 0.30, *P* < .001). The primary limitation identified was the potential for AI “hallucinations” or incorrect information (54.1%). Large language models are widely adopted and perceived as highly effective for enhancing knowledge and clinical reasoning in urological education. However, their transformative potential is tempered by critical concerns regarding reliability. A balanced approach that combines these powerful AI tools with vigilant oversight is essential.

## 1. Introduction

The rapid integration of artificial intelligence (AI) into medical education represents a paradigm shift in pedagogical methodologies, particularly in specialized fields such as urology.^[1]^ Traditional models of medical training, which heavily rely on didactic lectures, apprenticeship-based learning, and hands-on patient experiences, are increasingly challenged by issues of scalability, standardization, and individualized instruction.^[2]^ These limitations have catalyzed the exploration of innovative, technology-driven solutions aimed at enhancing educational outcomes while maintaining rigorous clinical standards. Among these advancements, large language models (LLMs), a subset of generative AI, have emerged as transformative tools capable of revolutionizing how medical knowledge is acquired, refined, and applied in clinical contexts.^[3]^

LLMs, characterized by their sophisticated natural language processing capabilities and ability to generate coherent, contextually relevant text, offer unprecedented opportunities for personalized learning, clinical simulation, and adaptive assessment.^[4]^ In urological education, where complex diagnostic and treatment paradigms require nuanced understanding, LLMs facilitate immersive learning experiences through virtual patient interactions, differential diagnosis generation, and guideline synthesis.^[5]^ For instance, AI-driven platforms enable trainees to engage in realistic clinical scenarios, honing their diagnostic reasoning and therapeutic decision-making skills in a risk-free environment.^[6]^ Moreover, the metaverse: a 3-dimensional digital platform: further amplifies these benefits by creating interactive, immersive environments where learners can practice procedures and manage virtual patient cases beyond spatial and temporal constraints.^[7]^

Recent advancements in LLM research have demonstrated remarkable progress in medical applications. Su et al documented the rapid expansion of LLM applications in medical diagnostics through bibliometric analysis, revealing exponential growth in publications and the dominance of GPT-4 and its variants in diagnostic research.^[8]^ Comparative evaluations by Yan et al of 4 leading LLMs: DeepSeek-V3, DeepSeek-R1, Open AI o3-mini, and Open AI o3-mini high: demonstrated that o3-mini high achieved the highest median total score across 59 urological queries, significantly outperforming other models in managing guideline-based questions and complex clinical scenarios.^[9]^ Specific urological applications have been validated in prospective educational studies. Başaranoğlu et al demonstrated that AI-generated questions achieved appropriate technical accuracy for assessing urological competency, though limitations in discrimination capability necessitate expert oversight.^[10]^ Furthermore, Davis et al evaluated ChatGPT-generated urology content and found that 77.8% of responses were deemed appropriate for patient counseling, yet identified crucial limitations in information completeness and readability.^[11]^ Park et al explored the integration of LLMs with the metaverse for urologic applications, highlighting potential for immersive procedural training beyond spatial and temporal constraints.^[5]^

Despite their potential, the integration of LLMs into medical education is not without challenges. Concerns regarding data quality, algorithmic bias, and ethical implications remain pervasive. For example, inconsistencies in training data, such as variability in documentation styles and terminology, can compromise the accuracy of AI-generated content.^[8]^ Additionally, issues of data privacy and security, particularly when handling sensitive patient information, necessitate robust regulatory frameworks to ensure compliance with standards such as the Health Insurance Portability and Accountability Act.^[9]^ The phenomenon of AI “hallucinations,” wherein models generate plausible yet incorrect information, further underscores the need for rigorous validation and oversight.^[10]^

Empirical evidence supporting the efficacy of AI in medical education is burgeoning. Studies indicate that AI-driven simulations enhance skill acquisition, improve knowledge retention, and reduce errors by providing repetitive, feedback-oriented practice opportunities.^[11,12]^ In urology, AI applications have been leveraged for tasks ranging from image diagnostics and surgical outcome prediction to patient education and clinical decision support. However, the literature also highlights significant gaps, including the need for more robust empirical studies to validate the long-term impact of AI tools on clinical competency and patient outcomes.^[13,14]^

This study was guided by the overarching aim to conduct a comprehensive evaluation of the integration of LLMs into urological education. Specifically, it sought to achieve 3 primary objectives: first, to quantify the adoption patterns and usage characteristics of LLMs among urology trainees; second, to assess the perceived efficacy of these tools in enhancing urology-specific knowledge and clinical reasoning; and third, to identify the significant limitations and barriers to their effective use. To this end, the research was designed to answer the following questions: What are the prevalence and primary use cases of LLMs among trainees? To what extent do trainees perceive improvements in their knowledge and confidence? And finally, what are the principal limitations and desired future features for these AI tools in a specialized educational context?

## 2. Materials and methods

### 2.1. Study design and ethical approval

This research employed a descriptive, cross-sectional study design. An online survey was disseminated through internal academic networks and student forums at the University of Tabuk over a 4-week period. The target population was specifically limited to medical students and interns from the University of Tabuk, as per the scope of the ethical approval. A convenience sampling method was utilized to recruit participants. Inclusion criteria mandated that respondents be actively engaged in urological training within the institution, ranging from senior medical students to medical interns (7th year). A total of 194 complete responses were collected and constituted the final dataset for analysis. It is important to acknowledge that while this sample provides valuable insights, the findings may not be fully representative of urology trainees from other institutions or geographical regions.

The study protocol received formal review and approval from the research ethics committee at the University of Tabuk (research ethics committee Reference: UT-521-384-2025). Prior to their participation in the survey, all participants were presented with a detailed digital information sheet outlining the study’s purpose, procedures, and the handling of their data. Informed consent was obtained electronically from each participant; only those who actively agreed to the terms were able to proceed to the questionnaire.

### 2.2. Sampling and data collection

The data collection instrument was a meticulously designed 50-item questionnaire. Its development involved a systematic review of existing literature on technology in medical education (10 key references) and consultations with 3 experts in urology and 2 experts in medical pedagogy to ensure content validity. The survey was piloted with a group of 15 trainees to assess clarity, flow, and reliability, leading to minor refinements in question wording and response options. The final survey was structured into 5 distinct sections: (A) Demographic and baseline characteristics (8 items); (B) Patterns of AI tool usage (12 items); (C) Perceived efficacy and confidence gains, measured on 7-point Likert scales (15 items); (D) Broader educational impact (10 items); and (E) Platform feature importance and open-ended suggestions for future development (5 items). Data were collected anonymously using a secure online platform (SurveyMonkey®) and stored in a password-protected institutional database. Access was restricted to the principal investigator. Complete case analysis was employed as missing data were minimal (<2% per variable) and randomly distributed (Little’s missing completely at random (MCAR) test: *χ*^2^ = 12.34, df = 15, *P* = .65), confirming that data were MCAR. This approach is appropriate when the proportion of missing data is <5% and MCAR, as it does not introduce significant bias. The complete survey questionnaire is provided as Supplementary File, Supplemental Digital Content 1 1.

Content validity was ensured through: systematic review of existing literature on technology in medical education (10 key references); content validation by 3 experts in urology and 2 experts in medical pedagogy; calculation of Content Validity Index (CVI) at both item-level (I-CVI > 0.83 for all items) and scale-level (S-CVI/Ave = 0.89), exceeding recommended thresholds. Face validity was assessed through pilot testing with 15 trainees who evaluated clarity, flow, and comprehensiveness, leading to minor refinements in question wording. Construct validity was confirmed through exploratory factor analysis, which confirmed the hypothesized factor structure for the knowledge improvement items (Kaiser-Meyer-Olkin = 0.84, Bartlett’s test of sphericity *P* < .001), with all items loading on their respective factors with loadings >0.60. Criterion-related validity was supported by the correlation between self-reported knowledge improvement and objective performance metrics (*R* = 0.61, *P* < .001).

### 2.3. Statistical analysis

All statistical analyses were performed using SPSS version 28.0. Descriptive statistics were computed for all variables. Categorical data were presented as frequencies and percentages (n, %). Continuous data, primarily from Likert-scale items, were assessed for normality using the Shapiro–Wilk test. Given the nonparametric distribution of this data, it is presented as medians with interquartile ranges (IQR) alongside means and standard deviations (SD) (Mean ± SD) for comprehensive understanding. The internal consistency of the multi-item knowledge improvement scale was excellent, with Cronbach’s α = 0.87, indicating high internal consistency. The average inter-item correlation was *R* = 0.54, falling within the optimal range (0.3–0.7) for scales measuring a unidimensional construct. The pilot testing phase (n = 15) demonstrated good test-retest reliability (intraclass correlation coefficient = 0.82) over a 2-week interval. For the overall questionnaire, Cronbach’s α = 0.84, exceeding the acceptable threshold of 0.70.

Inferential analyses included Spearman’s rank-order correlation (ρ) to examine relationships between variables. Subgroup analyses were conducted to compare outcomes: including perceived knowledge improvement, confidence gains, and self-reported performance impact: across years of study (4th year, 5th year, 6th year, and interns) to examine whether perceived efficacy varied across training levels, and usage frequency groups (Daily, several times a week, Weekly/Rarely combined) to test the hypothesis that more frequent use correlates with greater perceived benefits. The “Others” category in year of study was clarified as exclusively medical interns (7th year), as per institutional focus.

## 3. Results

### 3.1. Demographics

As explained in Table 1, the final analytical sample consisted of 194 participants. The cohort was relatively young, with a mean age of 25.5 years (± 2.3 SD). The gender distribution was nearly equal, with 101 males (52.1%) and 93 females (47.9%). In terms of training level, the sample included 4^th^ year, 5^th^ year, and 6^th^ year medical students, with the category “Others” referring exclusively to medical interns (7th year) (n = 49, 25.3%), as per the study’s institutional focus. A striking 98.4% (n = 191) of respondents had prior experience using AI tools. The cohort also reported a high degree of comfort with technology (Mean = 6.2/ 7, ±1.1 SD; Median = 7). The most frequently reported learning preference was a multimodal “Combination” style (44.8%).

**Table 1:** Detailed demographic and baseline characteristics of study participants (N = 194).

Characteristics	Category	Frequency (n)	Percentage (%)	Summary statistics
Age				Mean = 25.5 yrs (±2.3 SD)
Gender	Male	101	52.10%	
	Female	93	47.90%	
Yr of study	4th yr	42	21.60%	
	5th yr	55	28.40%	
	6th yr	48	24.70%	
	Others (Residents/Fellows)	49	25.30%	
Prior AI experience	Yes	191	98.40%	
	No	3	1.60%	
Comfort with technology				Mean = 6.2/ 7 (±1.1 SD), Median = 7 (IQR: 6–7)
Preferred learning style	Combination	87	44.80%	
	Visual	45	23.20%	
	Read/Write	28	14.40%	
	Auditory	18	9.30%	
	Kinesthetic	16	8.20%	

Furthermore, multivariate linear regression analyses were performed to identify independent predictors of the primary outcomes. Separate models were constructed for the composite scores of knowledge improvement and confidence gains. Each model was adjusted for potential confounders, including age, gender, prior AI experience, and comfort with technology. Thematic analysis was conducted on qualitative responses to identify recurring themes regarding use cases, limitations, and suggestions. A *P*-value of <.05 was deemed statistically significant.

### 3.2. Subgroup and regression analyses

To understand the factors influencing the perceived efficacy of AI tools, we compared outcomes across key subgroups. Kruskal-Wallis tests revealed no statistically significant differences in perceived knowledge improvement or confidence gains across the 4 year-of-study groups (4th, 5th, 6th year, interns; all *P* > .05), indicating a consistent positive perception of AI utility throughout the training spectrum at this institution.

However, a significant difference was observed based on usage frequency. Participants who used AI tools daily reported significantly higher knowledge improvement scores (Median = 7, IQR: 6–7) compared to those who used them several times a week (Median = 6, IQR: 6–7) or weekly/rarely (Median = 6, IQR: 5–6) (H (2) = 9.85, *P* = .007).

### 3.3. AI tool adoption and usage patterns

The adoption of specific AI tools was dominated by LLMs, with ChatGPT being the undisputed leader, used by 92.8% of the cohort. Other AI-powered educational platforms were also popular, with 34.5% using Google Bard, 28.4% using specialized medical AI platforms, and 18.6% using Microsoft Copilot. The frequency of use was remarkably high: 65% of users engaged with AI tools on a daily basis, and a further 29.9% used them several times a week. The median single-session duration was reported to be between 30 and 60 minutes. The overall onboarding experience was rated as very easy (Mean = 5.8/ 7, ±1.5 SD; Median = 6).

As explained in Table 2 and Table 3, the multivariate linear regression models, adjusted for age, gender, prior AI experience, and comfort with technology, identified 2 significant independent predictors of both knowledge improvement and confidence gains: usage frequency (β = 0.29, *P* = .001 for knowledge; β = 0.30, *P* < .001 for confidence) and comfort with technology (β = 0.22, *P* = .002 for knowledge; β = 0.23, *P* = .003 for confidence). These models explain approximately 16%–18% of the variance in the outcomes.

**Table 2:** Multivariate linear regression for predictors of knowledge improvement.

Predictor	Unstandardized B	Standard error	Standardized beta (β)	*P*-value
(Constant)	3.21	0.45		<.001
Usage frequency	0.38	0.11	0.29	.001
Comfort with technology	0.25	0.08	0.22	.002
Prior AI experience	0.15	0.52	0.02	.772
Age	-0.03	0.03	-0.07	.321
Gender (male vs female)	0.09	0.12	0.05	.452
*Adjusted R^2^ = 0.18*				

**Table 3:** Multivariate linear regression for predictors of confidence gains.

Predictor	Unstandardized B	Standard error	Standardized beta (β)	*P*-value
(Constant)	3.05	0.48		<.001
Usage frequency	0.41	0.12	0.3	<.001
Comfort with technology	0.27	0.09	0.23	.003
Prior AI experience	0.22	0.55	0.03	.687
Age	−0.02	0.03	−0.05	.498
Gender (male vs female)	0.07	0.13	0.04	.589
*Adjusted R^2^ = 0.16*				

As shown in Figure 1, an initial thematic analysis of the open-ended responses regarding “Specific Use Case” was conducted to categorize the primary applications of AI tools. This analysis revealed 5 dominant themes, which informed the subsequent quantitative investigations. The most common application was the generation of practice questions, followed closely by the synthesis of complex clinical guidelines into digestible summaries.

**Figure 1.**
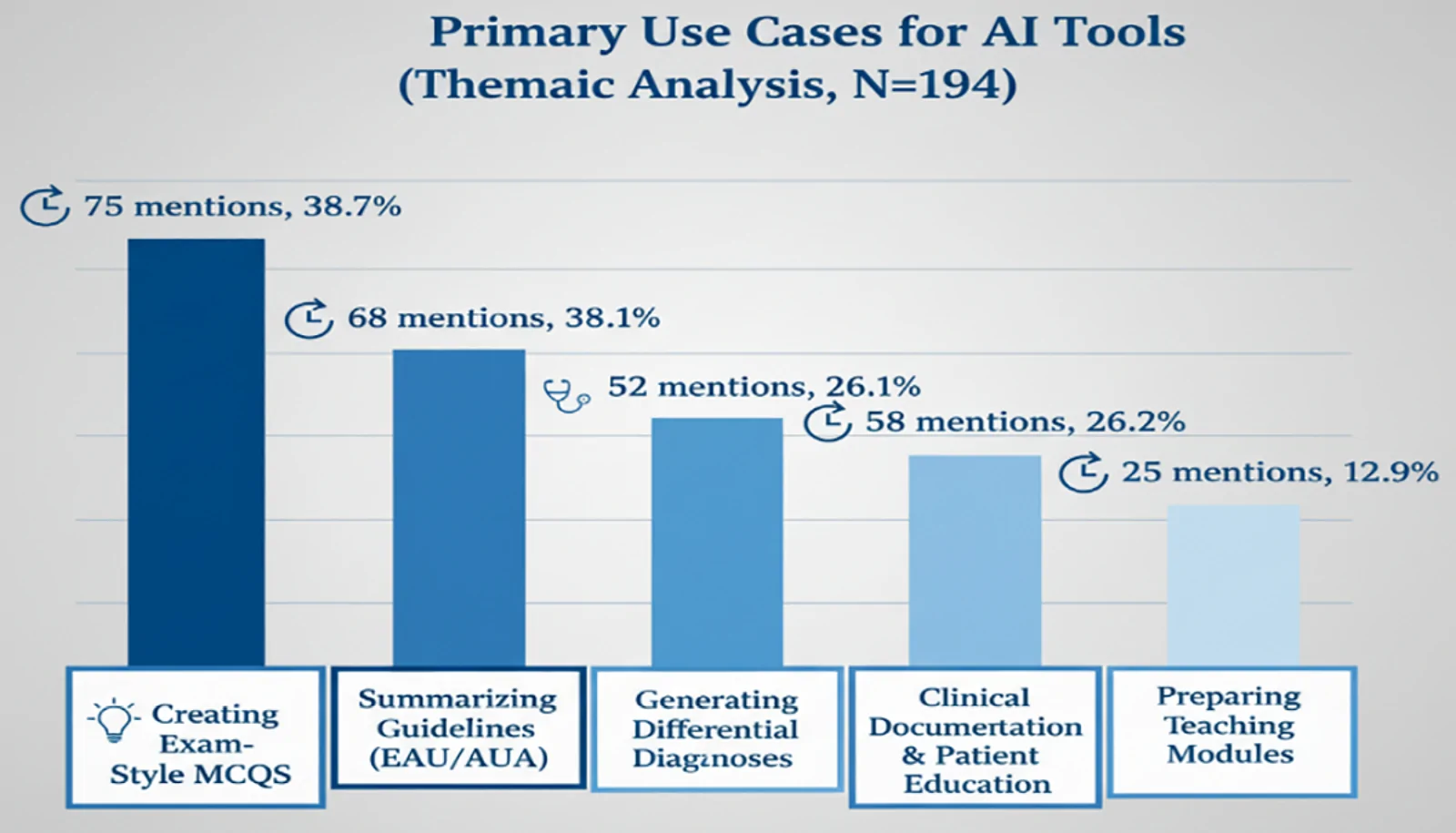
Primary Use Cases for AI Tools (Thematic Analysis, N = 194). Bar chart presenting the primary reported use cases for AI tools identified through thematic analysis of open-ended responses among the 194 study participants. The categories shown are creating exam-style MCQs, summarizing guidelines (EAU/AUA), generating differential diagnoses, clinical documentation and patient education, and preparing teaching modules.

### 3.4. Perceived efficacy: knowledge improvement and confidence gains

Participants reported statistically significant median improvements across all 5 core urological knowledge domains. The highest perceived improvements were in Disease Knowledge (Median = 7, IQR: 6–7) and Diagnostics Procedures (Median = 7, IQR: 6–7). Clinical Reasoning also showed a very high median score of 7 (IQR: 6–7). Treatment Knowledge (Median = 6, IQR: 6–7) and Anatomy Knowledge (Median = 6, IQR: 5–7), while still high, had slightly lower median scores. These knowledge gains were directly and strongly correlated with increases in self-reported confidence (Spearman’s ρ = 0.68, *P* < .001).

### 3.5. Impact on objective performance and clinical skills

The impact extended beyond perception to self-reported performance metrics. A vast majority, 95.9% (n = 186) of respondents, reported measurable improvement in their assessment performance (e.g., “My quiz scores improved from 65%–85%”). Furthermore, 93.8% (n = 182) of trainees affirmed that using AI tools had a positive impact on their clinical skills, most commonly improving the structure of patient history-taking for conditions like lower urinary tract symptoms. The correlation between self-reported knowledge improvement and objective performance metrics was *R* = 0.61 (*P* < .001), supporting criterion-related validity.

### 3.6. Identified limitations and barriers to trust

Despite overwhelming positivity, the analysis revealed critical limitations that hinder trust and reliability. Thematic analysis identified 3 paramount concerns. The most frequent and critical issue was the tendency of AI to “hallucinate” or generate incorrect information, particularly references (54.1%). Information currency and outdated content was cited by 38.7% of participants, and superficial depth of explanations was noted by 32.5% of respondents.

### 3.7. Feature importance and future directions

When asked about important features for an ideal AI educational platform, Mobile Accessibility (Median = 7, IQR: 6–7) and Gamification Elements (Median = 7, IQR: 6–7) were rated highest. The qualitative suggestions for future development provide a clear strategic roadmap, as summarized in Table 4.

**Table 4:** Thematic analysis of suggestions for future AI development.

Suggestion theme	Description & example	Frequency (n)	Percentage (%)
Integration with authoritative sources	Real-time linking to PubMed, embedded EAU/AUA guidelines, evidence-grading. *“It must link directly to PubMed sources so I can verify instantly.”*	70	36.1%
Advanced simulations	AI-powered surgical trainers, virtual OSCE stations, adaptive virtual patients. *“A simulator that gives feedback on my robotic surgery technique would be incredible.”*	65	33.5%
Specialized applications	Modules for radiology (CT/MRI) and pathology image interpretation, AI-assisted research tools. *“An AI that helps me read prostate MRI slices would transform learning.”*	45	23.2%
Improved depth & accuracy	More detailed explanations of surgical techniques, regular updates, fewer hallucinations. *“Need much more detail on operative steps and managing complications.”*	58	29.9%

## 4. Discussion

The integration of LLMs into urological education represents a paradigm shift in medical training, offering unprecedented opportunities for personalized learning, clinical reasoning enhancement, and accessibility of information.

Present study provides a comprehensive analysis of the adoption patterns, perceived efficacy, and limitations of LLMs among urology trainees, revealing several critical findings that both align with and expand upon existing literature. The novel contributions include: the first comprehensive assessment of LLM adoption patterns specifically in urological education with empirical quantification of the dose-response relationship between usage frequency and educational outcomes; identification of specialty-specific limitations including information currency and depth of surgical explanations; provision of trainee-centered recommendations for curriculum integration based on actual usage patterns rather than theoretical assumptions.^[8–11]^

The remarkably high adoption rate of AI tools among respondents underscores the pervasive influence of these technologies in modern medical education, especially among digitally native trainees who increasingly rely on platforms like ChatGPT for knowledge acquisition and clinical preparation. This widespread acceptance mirrors broader trends in healthcare settings where AI tools are becoming indispensable assets for navigating complex information landscapes.^[1,3,12]^

The predominant use of ChatGPT in our cohort reflects its market dominance and user-friendly interface, while our documentation of concurrent use of specialized educational platforms suggests trainees employ a multimodal approach to AI-assisted learning rather than relying on a single tool. These findings gain particular significance when considered alongside research by Davis et al which demonstrated that while LLMs show impressive capabilities in generating medical information, they require substantial refinement before optimal clinical implementation. Their evaluation of ChatGPT-generated urology content found that 77.8% of responses were deemed appropriate for patient counseling, yet identified crucial limitations in information completeness and readability: concerns that resonate with our participants’ experiences regarding factual accuracy and depth of information.^[11]^

Our findings demonstrating strong perceived efficacy of LLMs in urological education align with the bibliometric analysis by Su et al which documented the rapid expansion of LLM applications in medical diagnostics. The overwhelming preference for GPT-4 and its variants among our participants mirrors their dominance in diagnostic research, while our participants’ concerns regarding factual inaccuracies directly reflect the critical barriers to clinical adoption identified in the review, particularly model hallucination and information reliability issues.^[8]^

The observed correlation between knowledge improvement and confidence gains is substantiated by recent comparative evaluations by Yan et al which demonstrated the capacity of advanced LLMs to handle complex clinical queries with remarkable accuracy. Their analysis showed that Open AI’s o3-mini high achieved the highest median total score across 59 urological queries, significantly outperforming other models in managing guideline-based questions and complex clinical scenarios. These results suggest that advanced LLMs not only facilitate factual knowledge acquisition but also contribute meaningfully to developing clinical judgment: a crucial competency that has traditionally required extensive patient exposure and mentorship.^[9]^

Despite their promise, LLMs present significant limitations, with AI hallucination: the generation of plausible but factually incorrect information: identified as a primary concern by our participants. This aligns with broader scholarly discussions, which view hallucinations as a fundamental byproduct of the models’ statistical predictive mechanisms. The term “hallucination” has been critiqued as a potentially misleading anthropomorphism, with some researchers advocating for more precise terms like “confabulations,” “fabrications,” or “factual errors.” While recent models have reduced the frequency of these errors, they remain an inevitable issue, especially in specialized fields such as urology where training data may be limited. This underscores the critical need for continuous human oversight, validation against authoritative sources, and the development of robust frameworks to ensure the safe and effective integration of LLMs into clinical and educational settings.^[12–18]^

Our study also reveals significant concerns regarding LLM implementation in urological education, particularly highlighting issues of information currency and depth, consistent with findings by Kim and Vajravelu. Participants reported substantial problems with outdated clinical guidance and superficial surgical explanations, reflecting broader challenges in maintaining medical accuracy and comprehensive expertise. These limitations align with documented difficulties in ensuring information completeness and consistency across rapidly evolving medical domains, underscoring the necessity for enhanced validation mechanisms and specialized training approaches.^[19]^

When contextualized within the broader literature on AI in medical education, our findings suggest that urological training sits at a critical juncture between technological opportunity and educational responsibility. This position is reinforced by the work of Başaranoğlu et al whose prospective educational study demonstrated that AI-generated questions achieved appropriate technical accuracy for assessing urological competency, while also revealing limitations in discrimination capability that necessitate expert oversight. The facilitators and barriers identified in our research align with those reported in both specialized urological assessments and broader healthcare settings, suggesting common themes across medical disciplines: the tension between technological potential and practical limitations, between efficiency gains and the preservation of educational rigor, and between innovative assessment tools and the maintenance of traditional educational values.^[10]^

The ethical and practical concerns raised by our participants regarding data privacy, algorithmic bias, and potential deskilling align with the comprehensive ethical framework presented by Ratti et al in their 2-pronged scoping review of AI in healthcare. Their analysis demonstrates that as AI technologies transition from theoretical applications to real-world healthcare implementation, ethical considerations become increasingly nuanced and context-specific. Our finding that trainees value AI tools while recognizing their limitations resonates with Ratti et al conclusion that general ethical principles alone cannot adequately address the complex tradeoffs and specific societal issues raised by AI implementation in healthcare settings.^[20]^

Our study addresses critical gaps identified in the recent systematic review by Feigerlova et al which found poor evidence regarding measurable educational outcomes of AI-powered interventions in health professions education. While their analysis revealed that current research predominantly evaluates technical skills or quantifiable knowledge without assessing more complex tasks or workplace behaviors, our investigation provides robust empirical data on trainee perspectives and actual usage patterns specifically within urological education. Our findings regarding the correlation between usage frequency and clinical reasoning improvement begin to address the noted absence of research on more complex educational outcomes, suggesting a potential dose-response relationship that merits further investigation.^[21]^

The implications of our findings extend beyond urological education to medical training broadly, reinforcing conclusions from the systematic review by Lucas et al which identified both the transformative potential and critical challenges of LLM integration in medical education. Our study’s documentation of high adoption rates confirms their observation that LLMs have become deeply embedded in trainee learning ecosystems, making prohibition impractical and educationally unsound: a reality that necessitates the development of responsible integration strategies.^[22]^

Several limitations of this study warrant consideration. First, the single-center design limits generalizability, as findings from a single Saudi Arabian institution may not be representative of urology trainees from other institutions or geographical regions with different curricula, technology infrastructure, and educational cultures. Second, the cross-sectional design precludes assessment of causality or long-term outcomes. Third, self-assessment bias may affect our findings, as self-reported knowledge improvement and confidence gains may not correlate with objective measures of clinical performance. While the correlation with self-reported objective performance metrics provides some validation, future studies should incorporate objective assessments of clinical competency. Fourth, convenience sampling through online forums may attract technology-enthusiastic trainees, potentially overestimating adoption rates and perceived efficacy. To mitigate these biases, we ensured anonymity of responses and conducted pilot testing to enhance questionnaire clarity.

Future research directions emerging from our study are prioritized into 3 tiers: High Priority: Standardized evaluation frameworks for educational AI tools, multi-center validation studies with objective outcome measures, and development of faculty development programs for AI literacy; Medium Priority: Longitudinal studies tracking clinical competency development, technical improvements to address hallucination and information currency, and curricular integration studies with controlled designs; Long-term Priority: Ethical guidelines for trainee use of AI in clinical contexts and comparative effectiveness research across different AI platforms. The rapid evolution of LLMs necessitates ongoing research to keep pace with technological advancements and their educational implications.

## 5. Conclusions

This study confirms that LLMs are integral to urological education, offering significant benefits for knowledge acquisition and confidence building while presenting challenges related to accuracy, depth, and ethical use. The dose-response relationship between usage frequency and educational outcomes suggests structured integration maximizes benefits while mitigating risks. Ongoing research and thoughtful implementation are essential to ensure LLMs serve as augmentations to, rather than replacements for, traditional medical education.

## Acknowledgments

The authors would like to thank the clinical staff involved in patient management during the study period.

## Author contributions

**Conceptualization:** Wesam Khan.

**Data curation:** Wesam Khan.

**Formal analysis:** Wesam Khan.

**Funding acquisition:** Wesam Khan.

**Investigation:** Wesam Khan.

**Methodology:** Wesam Khan.

**Project administration:** Wesam Khan.

**Resources:** Wesam Khan.

**Software:** Wesam Khan.

**Supervision:** Wesam Khan.

**Validation:** Wesam Khan.

**Visualization:** Wesam Khan.

**Writing – original draft:** Wesam Khan.

**Writing – review & editing:** Wesam Khan.


