# Supplementary material for: The adoption, efficacy, and limitations of large language models in urological education: A cross-sectional survey
Source: Medicine (Baltimore). 2026 Oct 16;105(42):e51124. doi: 10.1097/MD.0000000000051124 (PMC13681085; doi:10.1097/MD.0000000000051124)
Supplement: Supplementary file 1 [file medi-105-e51124-s001.docx]

**COVER LETTER**

Date: September 24, 2026

To: The Editor-in-Chief
Medicine®
Wolters Kluwer

**Subject: Resubmission after technical formatting corrections – MD-D-25-14876R1**

Manuscript: “The Adoption, Efficacy, and Limitations of Large Language Models in Urological Education: A Cross-Sectional Survey”

Dear Editor,

We are pleased to resubmit the manuscript after completing the technical formatting corrections requested by the journal. The main manuscript now contains no tables, and the Figure 1 legend is placed at the end of the manuscript after the references. Each study table has been prepared as a separate individual Word document. Figure 1 is provided separately in journal-accepted image formats. Supplemental content will be uploaded as individual files using the OA_Supplemental Digital Content item type, as requested.

These changes are technical and organizational only and do not alter the study dataset, analyses, or substantive conclusions. A revision-marked manuscript and a clean manuscript are provided, together with the separate table and figure files and a point-by-point response to the current editorial queries.

Thank you for your consideration of our revised submission.

Yours sincerely,

Wesam Khan, MD
Corresponding Author
Department of Urology
University of Tabuk
Tabuk, Saudi Arabia
dr.khan_alfattani@hotmail.com
